# Imaging and microarray analyses of cell cycle arrest and apoptosis by Human T cell Leukemia Virus type 1 (HTLV-1) Tax

**DOI:** 10.1186/1742-4690-8-S1-A175

**Published:** 2011-06-06

**Authors:** Mariluz Arainga-Ramirez, Yoko Aida

**Affiliations:** 1 Medical Genome Sciences Department, The University of Tokyo, Tokyo, Japan; 2 Viral Infectious Diseases Unit, RIKEN, Saitama, Japan

## 

HTLV-1 Tax protein is an essential transcriptional positive regulator of viral expression, interacts with a number of cellular proteins and is a potent activator of viral and cellular gene expression. It has been previously shown that HTLV-1 infected cells arrest at the G1/S boundary when subjected to cellular stress. In addition, it has been observed HTLV-1 infected cells to undergo increased apoptosis upon cellular stress. In this study, we performed time-lapse imaging to explore the spatiotemporal patterns of cell-cycle dynamics during expressing Tax in fluorescent ubiquitination based cell-cycle indicator Fucci-Hela cells. In the case of Tax-expressing cells, we were able to monitor dynamics of cells arresting at the G1 phase and apoptotic cells, which showed rounded morphology and detached from dish after the cell cycle arrest. To elucidate the mechanism by which Tax induces the cell cycle arrest and apoptosis, we analyzed the regulation of a panel of host cellular genes by Tax using the microarray technology. We identified 17 Tax-dependent genes related to cell cycle regulation resulting in >2.0 fold up- or down-regulation and they were involved in response to stress and DNA damage, cell proliferation, mitotic cell cycle and inflammatory response. Additionally, 23 pro- and anti-apoptotic genes were regulated by Tax. Up-regulated genes were validated by real time RT-PCR analysis. To clarify the genes involved in Tax functions, we are conducting knockdown experiments for up-regulated genes. Collectively, our study permits the understanding of the biological events affected by retrovirus Tax, as regulation of cell cycle and apoptosis.

